# The effect of temperature on physical activity: an aggregated timeseries analysis of smartphone users in five major Chinese cities

**DOI:** 10.1186/s12966-022-01285-1

**Published:** 2022-06-14

**Authors:** Janice Y. Ho, William B. Goggins, Phoenix K. H. Mo, Emily Y. Y. Chan

**Affiliations:** 1 The Jockey Club School of Public Health and Primary Care, The Chinese University of Hong Kong, Hong Kong, China; 2 Nuffield Department of Medicine, University of Oxford, Oxford, UK

**Keywords:** Temperature, Physical activity, Step count, China, Urban

## Abstract

**Background:**

Physical activity is an important factor in premature mortality reduction, non-communicable disease prevention, and well-being protection. Climate change will alter temperatures globally, with impacts already found on mortality and morbidity. While uncomfortable temperature is often perceived as a barrier to physical activity, the actual impact of temperature on physical activity has been less well studied, particularly in China. This study examined the associations between temperature and objectively measured physical activity among adult populations in five major Chinese cities.

**Methods:**

Aggregated anonymized step count data was obtained between December 2017-2018 for five major Chinese cities: Beijing, Shanghai, Chongqing, Shenzhen, and Hong Kong. The associations of temperature with daily aggregated mean step count were assessed using Generalized Additive Models (GAMs), adjusted for meteorological, air pollution, and time-related variables.

**Results:**

Significant decreases in step counts during periods of high temperatures were found for cold or temperate climate cities (Beijing, Shanghai, and Chongqing), with maximum physical activity occurring between 16 and 19.3 °C. High temperatures were associated with decreases of 800-1500 daily steps compared to optimal temperatures. For cities in subtropical climates (Shenzhen and Hong Kong), non-significant declines were found with high temperatures. Overall, females and the elderly demonstrated lower optimal temperatures for physical activity and larger decreases of step count in warmer temperatures.

**Conclusions:**

As minor reductions in physical activity could consequentially affect health, an increased awareness of temperature’s impact on physical activity is necessary. City-wide adaptations and physical activity interventions should seek ways to sustain physical activity levels in the face of shifting temperatures from climate change.

**Supplementary Information:**

The online version contains supplementary material available at 10.1186/s12966-022-01285-1.

## Background

The public health burden of hot and cold temperatures has been documented by studies showing impacts on excess mortality [1–5], hospital admissions [3, 6–8]. However, much less has been discussed about the impact of temperatures at the level of daily human activity. Physical activity is an important factor for premature mortality reduction, as insufficient physical activity is attributed to be responsible for an estimated 9% of premature deaths worldwide [10]. Furthermore, physical activity plays an essential role in preventing non-communicable disease and enhancing well-being [11–13]. Poor weather has often been described as a ‘barrier to physical activity’ in qualitative studies [14, 15], and research has shown that objectively-measured physical activity can be affected by temperatures [14, 16–19]. However, few studies have been conducted in the Asia, particularly in China, although this relationship may vary across different climates and regional contexts.

With a population of 1.4 billion people as of 2019 [20], China currently has the largest population worldwide. Over the recent decades, the physical activity levels of the population have decreased, particularly in more urbanized areas [21]. A study found that work- and household- physical activity decreased by nearly half between 1991 and 2011, while active leisure and transport physical activity did not see a meaningful change in the same period [22]. According to World Health Organization (WHO) data, estimates of physical inactivity prevalence in 2016 among adults in China was 14.11% (10.1-19.37) [23]. The prevalence was found higher among Chinese people aged 45 and older in a nationwide survey, with physical inactivity prevalence at 19.31% (95% CI: 18.28–20.38%) in 2015 [24]. The Tsinghua-Lancet Commission on healthy cities in China calls for integration of health into all policies, including the increased facilitation of physical activity [21].

With climate change, temperatures in China are predicted to increase by 2.3 °C to 3.3 °C from 2000 to 2050 [25], increasing the occurrence of extremely hot temperatures in the coming decades. Yet there is a lack of understanding about the relationship between temperature and physical activity in China. Previous studies had only assessed small samples of the population [26, 27], or observed physical activity ecologically in specific locations such as public parks [28]. Ma et al. 2018 found a negative correlation between mean temperature and walking distance among 210 Pokemon GO players in Hong Kong [26]. Wang et al. 2017 reported no significant impact of temperature among 40 Beijing adults when monitored with an accelerometer every 2 months for an entire year [27]. Zhao et al. 2018 observed Harbin park users in the spring and found a positive correlation of ambient temperature with increased people conducting activities and increased intensity (METs) [28]. A more comprehensive understanding of temperature’s effect on physical activity is needed at the population level. This knowledge would support the development of policies to address these potential impacts and to promote physical activity in the cities, as called for in the Tsinghua-Lancet Commission.

Step counts are an important indicator of physical activity at the population level, as walking is a largely accessible, inexpensive, and a regularly conducted physical activity in everyday life [29]. The objective measurement of step counts has enabled studies to assess day-to-day variations of physical activity and its association with temperature [16, 19, 30–34]. However, studies have found different associations between temperature and step counts in various cities. Higher temperatures were associated with increased daily step count among COPD patients in London, United Kingdom [30] and residents in Prince Edward Island, Canada [32], whilst reduced steps were reported among adults in Qatar [16]. No associations were found between temperature and step counts among elderly in Cologne, Germany [33] and adults in Perth, Australia [31]. In Japan, curvilinear associations between temperature and step count were found among elderly in Nakanojo, with step counts peaking at 17 °C [19]. However, in Hokkaido, another Japanese study found elderly step counts were negatively associated with temperature during the snowfall season, but positively associated during non-snowfall season [34]. Multi-location comparisons could be useful to understand the varying associations between cities.

With the advancement of technology, step counts have been increasingly assessed using smartphone accelerometer applications, which have been highlighted for their convenience in population studies and validated for their accuracy [35, 36]. A systematic review found that the accuracy of smartphone physical activity measurements ranged from 73 to 100% regardless of phone placement (*n* = 10 studies), although lower accuracy was found for stair climbing (52-79% accuracy) [37]. Using smartphones enables utilization of the same objective physical activity measurement and study methodology across locations, facilitating multi-location comparisons [38–40].

This study examines the associations between mean temperature and daily step counts across five major Chinese cities.

## Methods

### Study setting and design

This is a prospective aggregated timeseries study. Five major Chinese cities were assessed including Beijing (located in the North), Shanghai (East), Chongqing (Southwest), Shenzhen (South), and Hong Kong SAR (South, Special Administrative Region). These are major cities in China, in terms of the population size, and economic and political significance (see Table 1). The cities are also located in four divergent areas of the country, with varying climates and topographies.

**Table 1:** Summary characteristics among five major Chinese cities (as of 2018)

	Overall China	Beijing	Shanghai	Chongqing	Shenzhen	Hong Kong
Population size (10,000 persons)	139,538	2154.2	2423.78	3101.79	1302.66	748.77
Area size (sq.km)	9,600,000	16,411	6340.50	82,403	1997.47	1081.8
Population density (person/sq.km)	148	1313	3823	390	6484	6890
Gross Domestic Product GDP (100 mil yuan)	900,309.5	30,320.0	32,679.87	20,363.19	24,221.98	34,273.79 (28,934.02 100 mil HKD)
GDP per capita (yuan)	64,644	140,211	134,982	65,933	189,568	459,989.2 (388,324 HKD)
Climate (Köppen-Geiger classification)	n/a	Dwa	Cfa	Cfa	Cwa	Cwa

Census data sources [41–46]; Köppen-Geiger classification [47]; HKD to CNY conversion (average rate of 2018): 1 to 0.8442 CNY, source: [48]

### Physical activity data

Anonymous aggregated secondary data on physical activity was obtained from the mobile application WeChat‘s in-app function WeRun (微信运动) for the duration of the study period. WeChat is an all-encompassing multi-function social media and messaging platform in China, with over 1.04 billion active monthly users as of 2018 [49]. The in-app function WeRun is a voluntary addition that enables users to compare fitness levels with their community and reads from the step count data of the phone’s health applications (iPhone or Android) or other data sources such as smartwatches, as allowed by the user. Both the iPhone and Android phones have been validated against regularly accepted pedometers and accelerometers in field-based research [35, 50]. These studies have found comparable estimates in both laboratory and free-living environments, although the mobile phone-proxy estimates may be liable to underestimation due to inconstant phone carrying [35, 50].

The secondary physical activity data was obtained from users who had specifically enabled the fitness tracking function of WeRun, authorizing the collection of their daily step counts. All data was anonymized prior to retrieval and only obtained in aggregate form to protect personal privacy. Aggregated mean daily step counts were obtained for each city from anonymized users of the in-app function who were located in the city at night (10 pm). Aggregated mean daily step counts were also obtained stratified by gender and age group (18-64, 65+) along with the number of anonymized users included in each aggregate value.

### Weather and pollutant data

Meteorological data were obtained from the China Meteorological Administration for the following stations for the mainland Chinese cities: Beijing (ID: 54511), Shanghai (ID: 58362), Chongqing (ID: 57516), and Shenzhen (ID: 59493). Data from Hong Kong was obtained from the Hong Kong Observatory. Daily mean temperature was used as the main exposure for this analysis, and also adapted into apparent temperature and percentile temperature of the study period. Apparent temperature was calculated from temperature and relative humidity using the following formulas [51, 52]:

$$H=\left(\log 10(RH)-2\right)/0.4343+\left(17.62\ast {T}_{air}\right)/\left(243.12+{T}_{air}\right)$$

$${T}_{dewpt}=243.12\ast H/\left(17.62-H\right)$$

$${T}_{apparent}=-2.653+0.994\left({T}_{air}\right)+0.0153{\left({T}_{dewpt}\right)}^2$$

where RH = relative humidity, T_air_ = air temperature, T_dewpt_ = dew point temperature, and T_apparent_ = apparent temperature. Other daily meteorological covariates obtained from the meteorological stations included: mean relative humidity, total rainfall, mean wind speed, mean atmospheric pressure, and total sunshine hours. A square root transformation was done for rainfall and windspeed, in order to reduce the effect of outliers. Extreme weather event information on typhoon days were incorporated as binary indicators from a WMO report on China and the Hong Kong Observatory [53–55]. The occurrence of super typhoon Mangkhut was included separately due to the severity of the storm, which made landfall in Shenzhen and Hong Kong on Sept 16, 2018.

Air pollution data was obtained from China National Environmental Monitoring Center network (CNEMC) and Hong Kong Environmental Protection Department. An air quality index was used instead of individual air pollutant variables, to reduce possible collinearity. In China, the Air Quality Index (AQI) is based on the concentration levels of six pollutants (SO2, NO2, PM2.5, PM10, CO, O3) and reported using a scale of 1-300+ [56]. All hourly AQI values were aggregated to the daily level and further log-transformed to adjust for the right skew. Missing variables were imputed using a simple moving average for consecutively missing data of twelve hours or less. Longer consecutive missing data were left as missing. The imputation was completed with the R package ‘imputeTS’. In Hong Kong, the Air Quality Health Index (AQHI) was used, based on the concentration levels of four pollutants (SO2,NO2, O3, and particulate matter) and reported using a scale of 1-10+ [57]. Hourly AQHI values were available for twelve general stations located throughout the city, which were averaged together to indicate the daily value for the entire city. For values ‘10+’, a value of 12 was used in the daily aggregation. The Tap Mun monitoring station was not included, as its rural location is not reflective of the residence of the general population.

Time-related variables, such as month, day of week (DOW), and public holiday, were included to control the analysis. Mainland China had extra workdays to compensate for extended holiday periods, which were adjusted for in the analysis as well [58]. A city-wide marathon was included as a special event in Hong Kong during the study period and adjusted for in the analysis [59]. The authors were unable to identify the occurrence of city-wide events in the other Chinese cities during the study period.

### Statistical analysis

The associations of temperature were assessed on aggregated mean daily step count, adjusted by other meteorological conditions, air pollution index, and time-related variables. A stepdown analysis was conducted separately for each city using Generalized Additive Models (GAMs). Meteorological covariates with the highest *p*-value were removed in each model, until no variables with *p*-value over 0.1 remained. The Akaike information criterion (AIC) was also compared between models to ensure the model quality did not decrease. Air pollution index and time-related variables were kept in the model as control variables. The full model had a formula as follows:

$$\begin{aligned}E\left( Daily\ mean\ step\ count\right)&= s(Mean\ temperature,k=4)+ s(Relative\ humidity,k=4)+ s(Precipitation,k=4)\\&+ s(Windspeed,k=4)+ s(Pressure,k=4)+ s(Sunshine,k=4)+s(AQI\ or\ AQHI,k=4)\\&+ factor(DOW)+ factor(Holiday)+ factor(Month)+ factor(Extra\ workdays)\\&+ factor(Typhoon)+ factor(Super\ typhoon)+ factor(Marathon)\end{aligned}$$

where s() indicates the smoothing function of continuous independent variables in R package “mgcv”. k indicates the basis dimension for the smooth, such that k-1 is the maximum degrees of freedom considered for the variable. factor() indicates the categorical independent variables. AQI or AQHI indicates the Air Quality Index (used in China) or Air Quality Health Index (used in Hong Kong). DOW indicates the day of week. Meteorological variables were excluded if irrelevant to the city.

The analysis was further stratified by gender and age group. Sensitivity analyses assessed the 1) effect of apparent temperature, 2) effect of percentile temperature, 3) removal of air pollution index, and 4) removal of outlier data caused by Typhoon Mangkhut in Shenzhen and Hong Kong. Statistical significance level was set at *p* ≤ 0.05. All analyses were conducted using R version 3.5.2 [60], using the package “mgcv” (Mixed GAM Computation Vehicle with Automatic Smoothness Estimation) [61].

### Ethics approval

Ethics approval was obtained from the Survey and Behavioural Research Ethics Committee of The Chinese University of Hong Kong (Date: August 13, 2018).

## Results

### Descriptive statistics

The study period was from Dec 6, 2017 to Dec 31, 2018. During the study period, daily temperatures averaged from 12.7 °C (±SD 12.1) in Beijing, to 23.5 °C (±SD 5.3) in Hong Kong. The average amount of anonymized users included in the aggregated data during the study period were 11.1 million for Beijing, 9.6 mil for Shanghai, 2.8 mil for Chongqing, 4.9 mil for Shenzhen and 0.4 mil for Hong Kong. Compared to census data, the study samples in all five cities had a comparable gender distribution with their respective general populations (see Table 2). However, the sample populations tended to be significantly younger than the general populations.

**Table 2:** Demographic comparison between sample population and city population of five Chinese cities (Unit 10,000 persons)

City	Sample			Population (2018)		*p*-value
	Category	Sample size (%)	±SD	Category	N (%)	
**Beijing**	**Total**	1108.9 (100.0)	167.3	Total	2154.2 (100.0)	
	**Gender**					
	Male	555.2 (50.1)	81.7	Male	1095.6 (50.9)	0.879
	Female	553.7 (49.9)	85.7	Female	1058.6 (49.1)	
	**Age**			Total 15+	1927.6	
	18-64	1095.2 (98.8)	163.4	15-64	1686.2 (87.5)	< 0.001*
	65+	13.7 (1.2)	4.0	65+	241.4 (12.5)	
**Shanghai**	**Total**	963.5 (100.0)	137.3	Total	1462.4 (100.0)^	
	**Gender**					
	Male	462.5 (48.0)	63.9	Male	724.1 (49.5)	0.761
	Female	501.0 (52.0)	73.4	Female	738.2 (50.5)	
	**Age**			Total 18+	1285.9	
	18-64	947.2 (98.3)	132.3	18-59	783.9 (61.0)	< 0.001*
	65+	16.3 (1.7)	5.1	60+	502.0 (39.0)	
**Chongqing**	**Total**	286.0 (100.0)	46.1	Total	3101.8 (100.0)	
	**Gender**					
	Male	131.4 (45.9)	21.1	Male	1563.4 (50.4)	0.368
	Female	154.6 (54.1)	25.1	Female	1538.4 (49.6)	
	**Age**			Total 15+	2572.3	
	18-64	282.3 (98.7)	44.9	15-64	2135.0 (83.0)	< 0.001*
	65+	3.6 (1.3)	1.2	65+	437.4 (17.0)	
**Shenzhen**	**Total**	489.8 (100.0)	66.6	Total	1302.7 (100.0)	
	**Gender**					
	Male	269.3 (55.0)	37.8	Male	707.8 (54.3)	0.893
	Female	220.5 (45.0)	28.9	Female	594.9 (45.7)	
	**Age**			Total 18+	\	
	18-64	487.4 (99.5)	66.2	18-64	\	NA
	65+	2.4 (0.5)	0.5	65+	\	
**Hong Kong**	**Total**	42.8 (100.0)	6.7	Total	748.77 (100.0)	
	**Gender**					
	Male	18.6 (43.5)	2.9	Male	342.23 (45.7)	0.659
	Female	24.2 (56.5)	3.8	Female	406.54 (54.3)	
	**Age**			Total 15+	663.89	
	18-64	42.5 (99.3)	6.6	15 – 64	533.36 (80.3)	< 0.001*
	65+	0.3 (0.7)	0.1	65+	130.53 (19.7)	

Chi-square test was used to measure the overall difference in proportion between the sample and 2018 population data. Population age group categories were based on the available census data per city; no age group data was found for Shenzhen. ^Age and sex breakdown were only available for the registered population in Shanghai. Census data sources: [42–44, 46, 62]

\ indicates the absence of data

* *p* ≤ 0.05 indicates significant difference

**Fig. 1:**
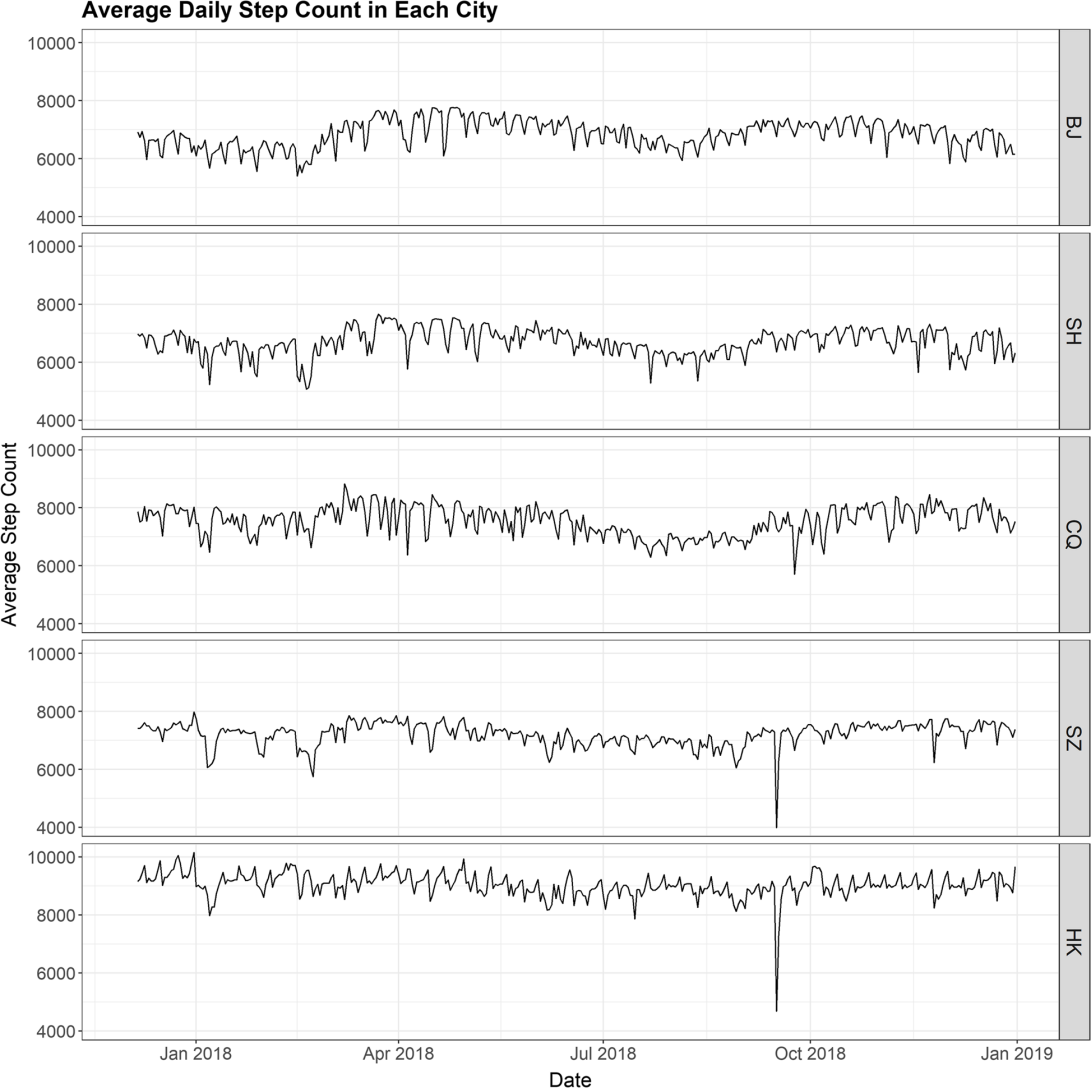
Trend of average daily step count in each city. Note: BJ = Beijing, SH = Shanghai, CQ = Chongqing, SZ = Shenzhen, HK = Hong Kong

The aggregated average step counts for each of the cities during the study period averaged 6846 steps for Beijing, 6703 for Shanghai, 7540 for Chongqing, 7209 for Shenzhen and 9040 for Hong Kong (see Table 3). The average step count was significantly higher among males than females (T-test, *p* < 0.001). Figure 1 demonstrates the trend of average daily step count during the study period for each city. The trends of average daily step count by gender and by age can be found in Supplemental materials, Figs. S1 and S2.

**Table 3:** Summary findings of five Chinese cities

Variables	Beijing (BJ)	Shanghai (SH)	Chongqing (CQ)	Shenzhen (SZ)	Hong Kong (HK)	***p***-value
Observation days	391	391	391	391	391	
**Physical activity**						
Total avg. daily step count, mean (±SD)	6846.0 (478.4)	6702.5 (463.3)	7540.1 (507.6)	7209.4 (402.7)	9039.6 (429.1)	< 0.001*
Males, mean (±SD)	7594.3 (518.2)	7465.3 (500.9)	8079.8 (461.9)	7862.8 (415.2)	9635.2 (430.4)	< 0.001*
Females, mean (±SD)	6095.1 (454.1)	5998.1 (444.3)	7081.4 (561.2)	6412.1 (416.4)	8580.1 (443.4)	< 0.001*
Age group 18-64, mean (±SD)	6850.0 (477.6)	6707.7 (461.2)	7535.6 (505.7)	7208.0 (401.8)	9047.7 (429.2)	< 0.001*
Age group 65+, mean (±SD)	6505.9 (614.4)	6385.3 (648.2)	7899.5 (708.8)	7485.1 (620.6)	7966.0 (461.9)	< 0.001*
**Meteorological**						
Station ID	54511	58362	57516	59493	HKO	
Temperature, range	−9.2 to 32.5	−1.0 to 32.6	4.5 to 36.5	6.6 to 30.8	9.0 to 31.2	
Temp, °C mean (±SD)	12.7 (12.1)	17.0 (9.3)	18.7 (8.4)	23.0 (5.5)	23.5 (5.3)	< 0.001*
Apparent temp, °C mean (±SD)	13.5 (12.6)	18.0 (12.5)	19.6 (11.1)	26.0 (8.7)	26.8 (8.3)	< 0.001*
Relative humidity, mean (±SD)	48.0 (19.2)	73.4 (12.6)	75.23 (12.0)	74.7 (14.0)	76.2 (10.8)	< 0.001*
Rainfall days, non-zero (%)	58 (14.8)	140 (35.8)	173 (44.2)	122 (31.4)	226 (57.8)	< 0.001*
Rainfall, mean (±SD)	1.4 (7.3)	3.7 (11.3)	3.1 (8.0)	5.0 (15.3)	5.5 (16.0)	< 0.001*
Windspeed, m/s (±SD)	2.0 (0.8)	2.6 (1.0)	1.3 (0.4)	1.9 (0.7)	6.6 (3.0)	< 0.001*
Pressure, mean (±SD)	1013.9 (10.6)	1016.8 (9.5)	983.6 (9.2)	1005.7 (7.0)	1013.1 (7.0)	< 0.001*
Sunshine, mean (±SD)	6.8 (3.7)	5.1 (4.2)	3.1 (4.1)	5.3 (3.8)	5.3 (3.9)	< 0.001*
**Precision variables**						
AQI, mean (±SD)	82.7 (48.7)	64.9 (32.7)	65.1 (31.5)	48.3 (17.0)	\	< 0.001*
AQHI, mean (±SD)	\	\	\	\	3.5 (1.1)	NA
Holiday (%)	25 (6.4)	25 (6.4)	25 (6.4)	25 (6.4)	19 (4.9)	0.863
Extra workdays (%)	7 (1.8)	7 (1.8)	7 (1.8)	7 (1.8)	0 (0.0)	NA
Typhoon (%)	0 (0.0)	5 (1.3)	0 (0.0)	2 (0.5)	9 (2.3)	0.001*
Super typhoon (%)	0 (0.0)	0 (0.0)	0 (0.0)	1 (0.3)	1 (0.3)	0.557
Marathon (%)	0 (0.0)	0 (0.0)	0 (0.0)	0 (0.0)	1 (0.3)	NA

Chi-square test was used to measure the overall difference in proportion between the cities

\ indicates the absence of data

* *p* ≤ 0.05 indicates significant difference

### Main models

The final models for Beijing, Chongqing, and Hong Kong underwent a stepdown process, in which atmospheric pressure and relative humidity were removed (see Table 4). For Shanghai and Shenzhen, no changes were required from the full models.

**Table 4:** Stepdown models of five Chinese cities

	Beijing (BJ)	Shanghai (SH)	Chongqing (CQ)	Shenzhen (SZ)	Hong Kong (HK)
	AIC (df)	AIC (df)	AIC (df)	AIC (df)	AIC (df)
Full model	4833.6 (34.0)	4851.0 (38.6)	4930.0 (39.7)	4795.1 (38.9)	5369.0 (32.6)
Stepdown 1	4828.1 (33.8)	Process stopped	4931.6 (38.2)	Process stopped	5367.1 (31.6)
Stepdown 2	Process stopped		Process stopped		5365.2 (30.6)
Variables removed	Removed pressure		Removed pressure		Removed pressure, RH

*AIC* Akaike information criterion, *df* degrees of freedom

**Fig. 2:**
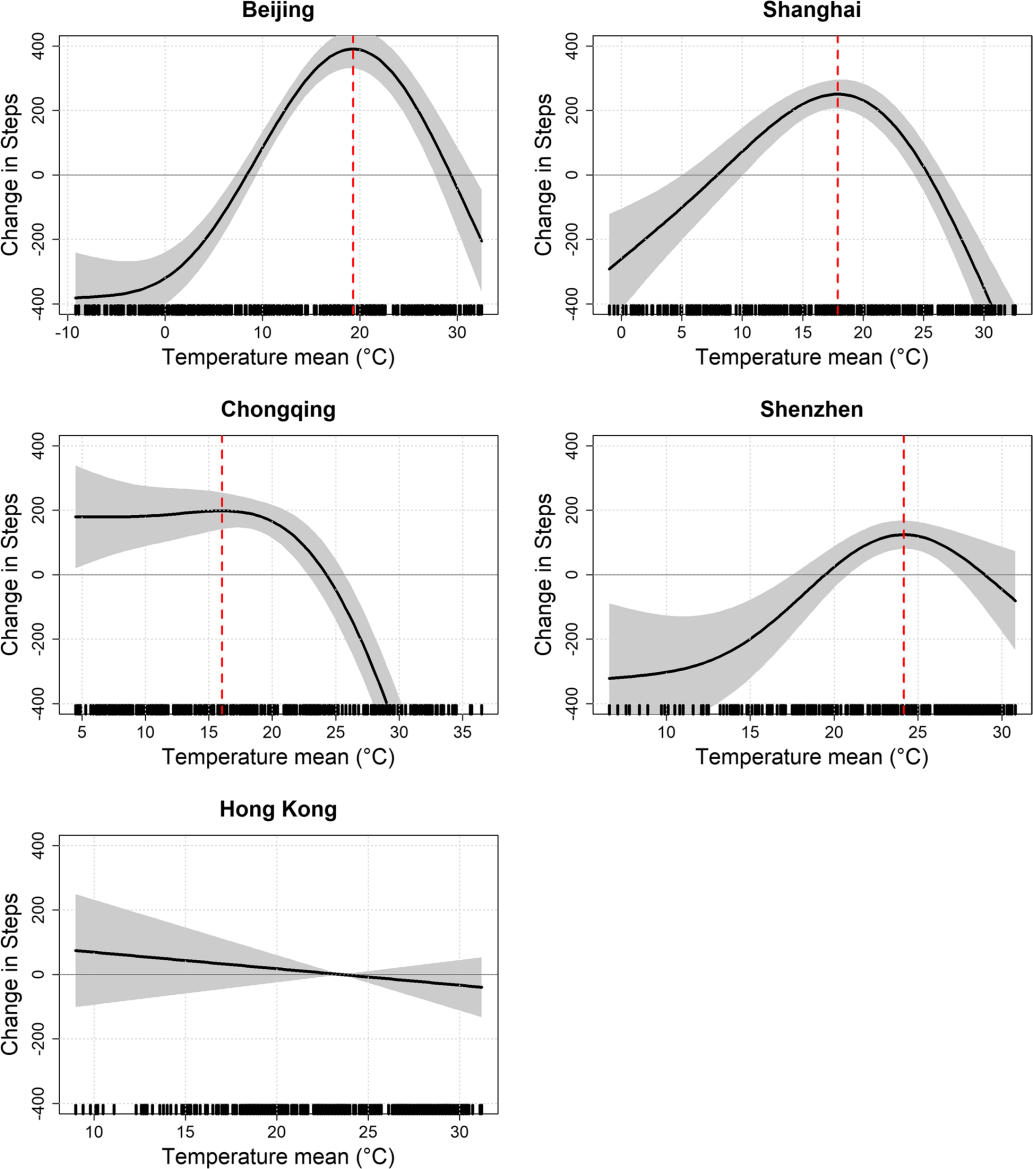
Relationships between temperature and daily step count in five Chinese cities. Note: The model for each city was adjusted for relative humidity#, precipitation, windspeed, pressure#, sunshine, AQI/AQHI, month, day of week, public holiday, extra workdays, typhoon, super typhoon, and marathon (#some cities had these variables removed in the stepdown process). Black markings along the x-axis indicate the actual existing temperature data of each city; Vertical red dotted lines indicate the identified optimal temperature; Grey shading indicates the 95% confidence interval

Overall, three of five cities (Beijing, Shanghai, and Chongqing) had significant inverse U-shaped associations between temperature and daily step count in high temperatures (see Table 5 and Fig. 2). During periods of high temperature, populations in Beijing, Shanghai, and Chongqing had significantly lower physical activity compared to optimal temperatures, while no significant associations were found in Shenzhen and Hong Kong. In periods of low temperatures, while populations in Beijing, Shanghai, and Shenzhen also found significantly lower step counts compared to optimal temperatures, the amount of decrease was less than in hot temperatures. The optimal temperature of peak step counts varied slightly between cities. In Beijing, the estimate of optimal temperature was at 19.3 °C, with a change in − 386.0 steps (95% CI: − 626.6, − 145.5) for a 10 °C increase from optimal temperature. In Shanghai, the optimal temperature was 17.9 °C, with a change in − 432.7 steps (95% CI: − 636.2, − 229.1) and in Chongqing, the optimal temperature was 16.1 °C, with a − 321.7 decrease (95% CI: − 526.6, − 116.8) in average step count for 10 °C increase from optimal temperature. On days with extremely hot temperatures, step counts decreased by − 820 steps at 32.6 °C in Shanghai and − 1494 steps at 36.5 °C in Chongqing, when compared to their respective optimal temperatures.

**Table 5:** Mean temperature associations on daily average step count, by city

City	Beijing	Shanghai	Chongqing	Shenzhen	Hong Kong
Optimal temperature	19.3	17.9	16.1	24.2	20 ^a^
Change in steps at OptT - 10 °C (95% CI)	− 342.8 * (− 452.2, − 233.4)	− 251.6 * (− 423.0, − 80.1)	− 19.1 (− 293.1, 254.9)	− 351.7* (− 614.8, − 88.6)	−3.0 (− 331.8, 325.8)
Change in steps at OptT + 10 °C (95% CI)	−386.0 * (− 626.6, − 145.5)	−432.7 * (− 636.2, − 229.1)	−321.7 * (− 526.6, − 116.8)	−204.8 ^b^ (− 514.5, 104.8)	−105.4 (− 268.5, 57.6)
N	366	366	366	361	391
Adjusted R squared	0.88	0.86	0.86	0.80	0.73
AIC	4828.1	4856.0	4931.6	4795.1	5365.2

*BJ* Beijing, *SH* Shanghai, *CQ* Chongqing, *SZ* Shenzhen, *HK* Hong Kong, *OptT* optimal temperature, *CI* confidence interval, *AIC* Akaike information criterion. The model for each city was adjusted for relative humidity#, precipitation, windspeed, pressure#, sunshine, AQI/AQHI, month, day of week, public holiday, extra workdays, typhoon, super typhoon, and marathon (#some cities had these variables removed in the stepdown process)

^a^Where association was not curvilinear, the optimal temperature was pre-set to 20 °C

^b^A change from 30.8 °C was used for Shenzhen, since this was the upper limit of temperature data in the city

* *p* ≤ 0.05 indicates significant difference

In Shenzhen, a curvilinear association was found albeit non-significant at higher temperatures. At the highest temperature in the dataset (30.8 °C), there was a non-significant decrease of − 204.8 step counts (95% CI: − 514.5, 104.8) compared to the optimal temperature (24.2 °C). On the other hand, a weak non-significant negative linear temperature association was found for Hong Kong (Change in steps at 10 °C from pre-set temperature 20 °C: -105.4; 95% CI -268.5, 57.6).

For other meteorological variables, higher relative humidity was negatively associated with step counts in Shanghai, Chongqing, and Shenzhen in a non-linear manner (see Supplemental materials, Fig. S3). High relative humidity in Beijing had a non-significant association with average step count. Rainfall and windspeeds were negatively associated with daily step count in all five cities, while daily sunshine hours were positively associated with step count, with a particularly strong association observed in inland Chongqing. Where atmospheric pressure remained in the model, it was found to be positively associated with step counts in Shenzhen and Shanghai. The air pollution index was significantly associated with physical activity levels in all cities except Beijing. Overall, the final model of these cities explained 73 to 88% of the variance in daily mean step counts (see Table 5 for model information).

### Stratified analyses

When stratified by gender, a lower optimal temperature was found among females than males in all four cities with curvilinear associations (Beijing, Shanghai, Chongqing, and Shenzhen) (see Table 6 and Supplemental materials Fig. S4). A slightly larger decline in step counts was found in Beijing among females at 10 °C above the optimal temperature (28.7 °C, change in steps: -405.4; 95% CI: − 641.1, − 169.6). Alternately, in Shenzhen a slightly larger effect was found among females at 10 °C colder temperatures from optimal (13.5 °C, change in steps: -338.1; 95% CI: − 629.9, − 46.4). In Hong Kong, the associations among both males and females remained non-significant.

**Table 6:** Stratification results of the temperature-physical activity associations in five Chinese cities

City	Stratification	OptT^a^	OptT – 10 °C	Change in steps	95% CI	Sig.	OptT + 10 °C	Change in steps	95% CI	Sig.
BJ	Male	20.0	10.0	− 344.9	− 453.2, − 236.6	*	29.9	− 353.4	−614.0, − 92.7	*
	Female	18.7	8.7	− 339.2	− 456.4, − 222.0	*	28.7	−405.4	−641.1, − 169.6	*
	18-64	19.3	9.3	− 344.1	−453.0, − 235.2	*	29.3	− 383.8	− 624.1, − 143.5	*
	65+	16.5	6.5	− 314.9	− 517.0, − 112.8	*	26.5	− 466.4	− 762.7, − 170.1	*
SH	Male	18.6	8.6	− 249.6	− 422.6, −76.5	*	28.6	− 427.4	− 656.2, − 198.7	*
	Female	17.3	7.3	− 250.6	−427.5, −73.7	*	27.3	− 418.7	− 609.1, − 228.4	*
	18-64	18.0	8.0	−251.9	−422.1, −81.8	*	28.0	−430.1	−635.1, − 225.1	*
	65+	13.5	3.5	− 318.6	− 657.2, 20.1		23.5	− 501.3	−708.4, − 294.2	*
CQ	Male	18.0	8.0	16.1	−200.4, 232.6		28.0	− 336.2	− 551.6, −120.9	*
	Female	15.6	5.6	−76.6	−383.3, 230.1		25.6	− 376.7	− 589.0, − 164.5	*
	18-64	16.2	6.2	−20.4	− 289.2, 248.4		26.2	− 327.2	− 533.0, − 121.4	*
	65+	20^a^	10.0	−2.4	− 292.9, 288.2		30.0	− 1541.8	− 1927.0, − 1156.7	*
SZ	Male	24.3	14.3	− 291.8	− 536.9, −46.6	*	30.8^b^	− 185.8	− 486.0, 114.5	
	Female	23.5	13.5	−338.1	−629.9, −46.4	*	30.8^b^	− 242.2	− 571.0, 86.5	
	18-64	24.2	14.2	− 350.8	− 613.5, −88.0	*	30.8^b^	− 203.5	− 513.1, 106.0	
	65+	22.5	12.5	− 487.5	− 903.1, −71.9	*	30.8^b^	− 462.4	− 901.8, −23.0	*
HK	Male	20^a^	10.0	−4.5	− 312.7, 303.7		30.0	− 128.9	− 281.8, 24.1	
	Female	20^a^	10.0	−3.4	− 358.4, 351.6		30.0	−96.7	− 272.9, 79.5	
	18-64	20^a^	10.0	−3.6	− 333.1, 325.8		30.0	−104.0	− 267.5, 59.5	
	65+	17.1	9.0 ^b^	−41.6	− 348.9, 265.7		27.1	− 193.2	− 290.1, −96.4	*

*BJ* Beijing, *SH* Shanghai, *CQ* Chongqing, *SZ* Shenzhen, *HK* Hong Kong, *OptT* optimal temperature, *CI* confidence interval. The model for each city was adjusted for relative humidity#, precipitation, windspeed, pressure#, sunshine, AQI/AQHI, month, day of week, public holiday, extra workdays, typhoon, super typhoon, and marathon (#some cities had these variables removed in the stepdown process)

^a^Where association was not curvilinear, the optimal temperature was pre-set to 20 °C

^b^The upper or lower limit of temperature was reached for that city’s dataset. The upper limit of temperature in the Shenzhen dataset was at 30.8 °C. The lower limit of temperature in Hong Kong was at 9.0 °C

* *p* ≤ 0.05 indicates significant difference

When stratified by age group, a lower optimal temperature was also found for the elderly over 65 in all cities with curvilinear associations (Beijing, Shanghai, Shenzhen, Hong Kong) (see Table 6 and Supplemental materials Fig. S4). In Chongqing, the association among the elderly was no longer inverse U-shaped, but had a steep significant negative slope. Additionally, in warmer temperatures, the elderly were associated with a markedly larger decrease in step counts compared with the adult age group (aged 18-64), with an approximate additional reduction of ~ 70 steps in Beijing and Shanghai and ~ 1200 steps in Chongqing. Furthermore, in Shenzhen and Hong Kong, the association of decreased step counts in warm temperatures was found significant among the elderly, while still remaining non-significant among the adults. In cold temperatures, there was no clear difference between the elderly and adults in most cities, except in Shenzhen, where the elderly were associated with a larger decrease in step counts with an approximate additional 130 ~ steps.

### Sensitivity analyses

Several sensitivity analyses were conducted including 1) examining the effect of apparent temperature and 2) examining the effect of percentile temperature, 3) removal of air pollution index, and 4) removal of outlier data caused by Typhoon Mangkhut in Shenzhen and Hong Kong on Sept 16, 2018. The results were largely consistent with the primary findings (see Table 7).

**Table 7:** Sensitivity analyses of the temperature-physical activity associations in five Chinese cities

City	Model	OptT^a^	OptT - 10 °C	Change in steps	95% CI	Sig.	OptT + 10 °C	Change in steps	95% CI	Sig.	df	AIC
BJ	Original (Mean Temp)	19.3	9.3	−342.8	−452.2, −233.4	*	29.3	−386.0	−626.6, −145.5	*	33.8	4828.1
	Apparent Temperature	22.1	12.1	−250.2	−362.7, −137.7	*	32.1	−344.7	− 610.3, − 79.2	*	33.3	4836.1
	Percentile Temp	68th	10th	− 767.1	− 965.4, − 568.7	*	90th	−353.8	− 581.8, − 125.7	*	34.8	4814.3
	Without pollution index	18.8	8.8	− 310.5	− 419.7, −201.3	*	28.7	− 377.0	−613.1, −140.9	*	31.9	5161.6
SH	Original (Mean Temp)	17.9	7.9	−251.6	−423.0, −80.1	*	27.9	−432.7	−636.2, − 229.1	*	38.6	4851.0
	Apparent Temperature	18.6	8.6	−145.2	− 297.8, 7.4		28.6	− 187.3	−362.5, −12.1	*	36.6	4878.3
	Percentile Temp	54th	10th	− 356.9	−571.7, − 142.0	*	90th	−514.0	− 744.1, − 283.9	*	37.5	4845.4
	Without pollution index	17.9	7.9	− 237.9	− 408.2, −67.5	*	27.9	−402.2	− 597.9, − 206.5	*	35.0	5193.6
CQ	Original (Mean Temp)	16.1	6.1	−19.1	−293.1, 254.9		26.0	−321.7	−526.6, −116.8	*	38.2	4931.6
	Apparent Temperature	15.3	5.3	1.8	− 253.6, 257.1		25.3	− 138.3	− 319.8, 43.2		35.8	4984.7
	Percentile Temp	48th	10th	−51.3	− 284.2, 181.5		90th	− 812.0	− 1080.0, − 544.0	*	38.3	4949.1
	Without pollution index	19.3	9.3	− 126.1	− 398.6, 146.4		29.3	−501.0	− 808.0, − 194.0	*	36.4	5273.9
SZ	Original (Mean Temp)	24.2	14.2	−351.7	−614.8, −88.6	*	30.8	−204.8	−514.5, 104.8		38.9	4795.1
	Apparent Temperature	27.0	17.0	−96.0	− 274.8, 82.9		37.0	−126.5	−367.8, 114.7		36.2	4832.2
	Percentile Temp	58th	10th	− 279.7	− 509.9, −49.5	*	90th	− 171.8	− 390.3, 46.7		38.0	4794.8
	Without Pollution index	23.8	13.8	−310.1	−577.8, −42.5	*	30.8	− 213.4	−511.8, 85.2		36.1	5137.4
	Without Typhoon	24.2	14.2	− 352.6	−613.6, −91.6	*	30.8	−204.6	−513.2, 104.0		37.9	4781.1
HK	Original (Mean Temp)	20^a^	10.0	−3.0	−331.8, 325.8		30.0	−105.4	−268.5, 57.6		30.6	5365.2
	Apparent Temperature	20^a^	10.0	−9.5	−273.0, 254.1		30.0	−83.7	−150.4, −17.1	*	30.7	5365.0
	Percentile Temp	50th^a^	10th	−10.4	−221.3, 200.6		90th	−173.1	− 382.9, 36.8		30.7	5363.3
	Without Pollution index	21.9	11.9	−128.8	− 523.0, 265.3		31.2	−348.0	−697.8, 1.8		34.7	5373.7
	Without Typhoon	20^a^	10.0	−3.7	− 328.9, 321.6		30.0	−104.2	− 261.1, 52.7		29.9	5350.4

*BJ* Beijing, *SH* Shanghai, *CQ* Chongqing, *SZ* Shenzhen, *HK* Hong Kong, *OptT* optimal temperature, *CI* confidence interval, *df* degrees of freedom, *AIC* Akaike information criterion. The model for each city was adjusted for relative humidity#, precipitation, windspeed, pressure#, sunshine, AQI/AQHI, month, day of week, public holiday, extra workdays, typhoon, super typhoon, and marathon (#some cities had these variables removed in the stepdown process). Percentile temperatures were set to 10th and 90th percentiles for analysis

^a^Where association was not curvilinear, the optimal temperature was pre-set to 20 °C or 50th percentile

*p ≤ 0.05 indicates significant difference

For apparent temperature models, the AIC was higher than for the original models in all cities aside from Hong Kong. A slightly higher optimal apparent temperature was found in Beijing, Shanghai, and Shenzhen. A slightly lower optimal apparent temperature was found in Chongqing, although the effect at + 10 °C was no longer significant. In Hong Kong, the effect at + 10 °C from 20 °C was to significantly decrease step counts by − 83.7 (95% CI: − 150.4, − 17.1).

Optimal percentile temperature was found at the 48th percentile in Chongqing, 54th percentile in Shanghai, 58th percentile in Shenzhen, and 68th percentile in Beijing. Similar to the main model, no optimal percentile temperature was found for Hong Kong. The model AIC improved when using percentile temperature for all cities except Chongqing.

Without the pollution index, the results remained consistent in Beijing, Shanghai, and Shenzhen, although the model AIC had a substantial increase from each city’s original model. In Chongqing, the optimal temperature increased from 16 °C to 19.3 °C. Additionally, a curvilinear association was found in Hong Kong, with optimal temperatures at 21.9 °C and a marginally significant decrease of − 348.0 (95% CI: − 697.8, 1.8) for a 10 °C increase from optimal temperature.

The results remained consistent when removing the typhoon outlier for Shenzhen and Hong Kong, while the model AIC improved from the original. When the two cities were hit by Typhoon Mangkhut on Sept 16, 2018, the aggregated daily step counts on that date dropped significantly to 3992 and 4682, respectively compared to average step counts.

## Discussion

Inverse U-shaped associations of temperature on city-wide aggregated step counts in four of five Chinese cities (Beijing, Shanghai, Chongqing, and Shenzhen) were found, with significant decreases in high temperatures for three cities (Beijing, Shanghai, and Chongqing). Step counts peaked at optimal temperatures ranging from 16.0 °C in Chongqing, 17.9 °C in Shanghai, 19.3 °C in Beijing, to 24.2 °C in Shenzhen. In warm temperatures, average decreases of 322 to 433 steps were found for those cities at 10 °C increase from optimal temperature, while temperatures in Shenzhen did not extend high enough to find a significant association. On days with extremely hot temperatures, the mean step counts of the city population decreased as far as 800 to 1500 steps compared to the optimal temperature. The impact of temperature seemed to be greater in climates with wider temperature ranges, whereas cities in subtropical climates did not have significant declines in step counts on days with high temperatures. In Hong Kong, a non-significant association was found between temperature and step count, however, a marginally significant curvilinear association was found with optimal temperatures at 21.9 °C when the city-specific air pollution index (AQHI) was taken out of the model, and a significant negative association was found in high apparent temperatures. Optimal percentile temperatures ranged between 48th percentile in Chongqing to 68th percentile in Beijing. Other results remained largely consistent in the sensitivity analyses.

Only a few temperature-physical activity studies have previously been conducted in China or in the Asian region. Two studies located in Japan had similarly found curvilinear associations between temperature and step counts, with step counts peaking between 17 °C and 20.7 °C [19, 63]. A study from Harbin, China, a city in the far north with a very cold climate, found a positive association during the spring months between temperature and the intensity of activity and number of active persons in the public park [28]. A previous study in Beijing found no seasonal variation and hourly association between temperature and average physical activity among 40 Chinese participants of an accelerometer study [27]. However, the study seemed to only consider the possibility of a linear association using general linear models. In Hong Kong, a study on Pokémon Go users found a significant negative association between temperature and daily distance travelled in the summer [26], while the negative association in our study was non-significant.

In other published multi-location studies, a trail study across the USA found increasing optimal temperatures with warmer American-centric climate regions [18]. In this study, locations with similar climates had similar associations, however warmer locations did not necessarily have the greater effects in high temperatures. Chongqing and Shanghai (both climate Cfa under the Köppen-Geiger classification [47]) had similar optimal temperature peaks and clear decreased physical activity associations in warm temperatures. Warmer Shenzhen and Hong Kong (both climate Cwa) found similar optimal temperature peaks ranging in the early 20s °C, particularly in the Hong Kong model without air pollution. However, non-significant decreases in step counts were found with higher temperatures, as both cities had lower extreme temperatures (maximum temp: 30.8 °C and 31.2 °C, respectively) compared to the other cities. Surprisingly, Beijing (climate Dwa) had a relatively high optimal temperature (19.3 °C) and the highest percentile optimal temperature at 68th percentile, despite having an overall colder climate.

This study found that optimal temperatures for physical activity ranged between 48th to 68th percentile, with the highest percentile found in Beijing. As indicated by Beijing’s climate classification Dwa, the ‘a’ demonstrates hot summer temperatures in an overall cold snow climate zone ‘D’. This produces a wider temperature range than other cities, as seen in Table 3. With half of days having mean temperatures below 12 °C in Beijing, the population may seek to take advantage of the warmer temperatures and other connected weather conditions (increased daylight hours, absence of icy surfaces etc.) to conduct more active leisure activities [64], leading to the findings of a greater increase of physical activity in warmer temperatures and higher optimal temperature than other cities. Additionally, the inter-city variations in physical activity patterns seem to demonstrate population adaptation to local climates [65] and may have also been influenced by variations in infrastructural or spatial patterns of the urban environment, such as the city density and urban sprawl [66]. As mentioned in a Beijing study on travel behaviour, the built environment can significantly affect people’s allocation of time and pursuit of activities [67]. Other potential confounding factors such as socio-economic status, education, and employment may have also affected the analysis and the comparison across cities.

In the stratified analyses, lower optimal temperatures were found among females in all cities with curvilinear relationships. This is a new finding, as previous studies that stratified by gender did not assess for a difference in optimal temperature between genders [63, 64, 68, 69]. Only one study stratifying by gender found overall lower step counts among females compared to males [63], while other studies did not find clear differences in temperature-related physical activity between males and females [64, 68, 69]. Our study also found that elderly over 65 had lower optimal temperatures and larger decreases of step count in warmer temperatures compared with the adult age group. These findings are consistent with several previous studies that stratified by age and found stronger temperature effects among those over 65, particularly over 80 [63, 70, 71]. These are also aligned with the physiological understanding of a lower heat tolerance among older adults due to a decreased capacity to thermoregulate [72, 73].

### Strengths and limitations

This was the first multi-location comparative study on temperature and physical activity located in China and in Asia. This study demonstrated a decrease of daily physical activity in high temperatures using aggregated objectively measured step counts from a large, anonymized sample size. The data collection method ensured that anonymized users were located in the respective cities in order to be included in the analysis. A non-linear statistical analysis allowed for flexible associations between temperature and physical activity, as well as all other meteorological variables. The analysis comprised over a year’s duration, covered all seasons, and controlled for time-related variables and holidays and special events (typhoons and marathons) where feasible.

However, this study’s data collection was limited to only those who voluntarily downloaded the mobile application, and may have skewed towards the health-conscious, able-bodied (no mobility problems), younger, and more active subset of the population. This can be seen by the relatively high average daily step count of each city and the skewed age distribution compared to the general population, and suggests that this study may underestimate the temperature effect, particularly among more vulnerable populations. The accelerometer could only collect information on ambulatory activities when the phone was located with the person and was unable to account for any cycling or aquatic activities. As this study could not control for whether the anonymized users kept their phones on them, the aggregation from a large data sample could be an underestimation of actual physical activity levels. The aggregated data could not control at the individual participant level and would have included any visitors or temporary stay individuals who used the in-app function and were located in the city during any evenings of the study period. No uncertainty boundaries were provided around the step count estimates. Other potential confounding factors may not have been addressed in this study, such as socio-demographic characteristics, accessibility of transportation options, or indoor/outdoor activity. The effect of weather alerts and warnings on physical activity levels could not be adjusted in this analysis.

### Future research directions

Future studies should assess the temperature-physical activity relationship in more climate and geographically diverse locations of China and other regions. An increased understanding is needed on the role of urban planning and spatial patterns in affecting the relationship between temperature and physical activity. Extreme temperature events could be assessed in warmer subtropical climate locations like Shenzhen and Hong Kong, to elucidate the effect of extreme temperatures. Furthermore, the singular effect of the super typhoon in these two cities also hints at the large impact that extreme weather events can have on population physical activity patterns. With climate change and an increased frequency of heat waves, typhoons, storms, and other climate-related hazards, there may be increased days where population activity is lowered by such extreme events. Studies could further examine and project the impact of physical activity in extreme weather events.

## Conclusions

Temperatures and physical activity demonstrated inverse-U shaped associations among several major cities in China, with physical activity peaking between 16 °C to 19.3 °C in temperate climate cities. Coupled with rising temperatures due to climate change, these reductions in physical activity could subsequently lead to consequential health effects, as the temperatures shift upward to levels higher than the optimal temperature for physical activity. Adaptations to temperature should be addressed in China’s physical activity promotion guidelines, with healthcare providers empowered to provide appropriate physical activity recommendations and heat health prevention measures. Recommended city-wide interventions include increased access to indoor recreational facilities and urban design measures to alleviate the heat and to support sustainable physical activity levels in the face of climate change.

## Supplementary Information

**Additional file 1.**

## Data Availability

The data that support the findings of this study are available from WeChat WeRun (https://www.wechat.com/) but restrictions apply to the availability of these data, which were used under agreement for the current study, and so are not publicly available.
