# Supplementary material for: The effect of temperature on physical activity: an aggregated timeseries analysis of smartphone users in five major Chinese cities
Source: Int J Behav Nutr Phys Act. 2022 Jun 14;19:68. doi: 10.1186/s12966-022-01285-1 (PMC9195465; doi:10.1186/s12966-022-01285-1)
Supplement: Supplementary file 1 — Additional file 1. [file 12966_2022_1285_MOESM1_ESM.docx]

**Title: The effect of temperature on physical activity: An aggregated timeseries analysis of smartphone users in five major Chinese cities**

Authors: Janice Y. Ho, William B. Goggins, Phoenix K. H. Mo, Emily Y. Y. Chan

**Supplemental materials: Figures S1-4**

**Figure S1. Trend of average daily step count in each city, by gender**

Note: BJ = Beijing, SH = Shanghai, CQ = Chongqing, SZ = Shenzhen, HK = Hong Kong.

**
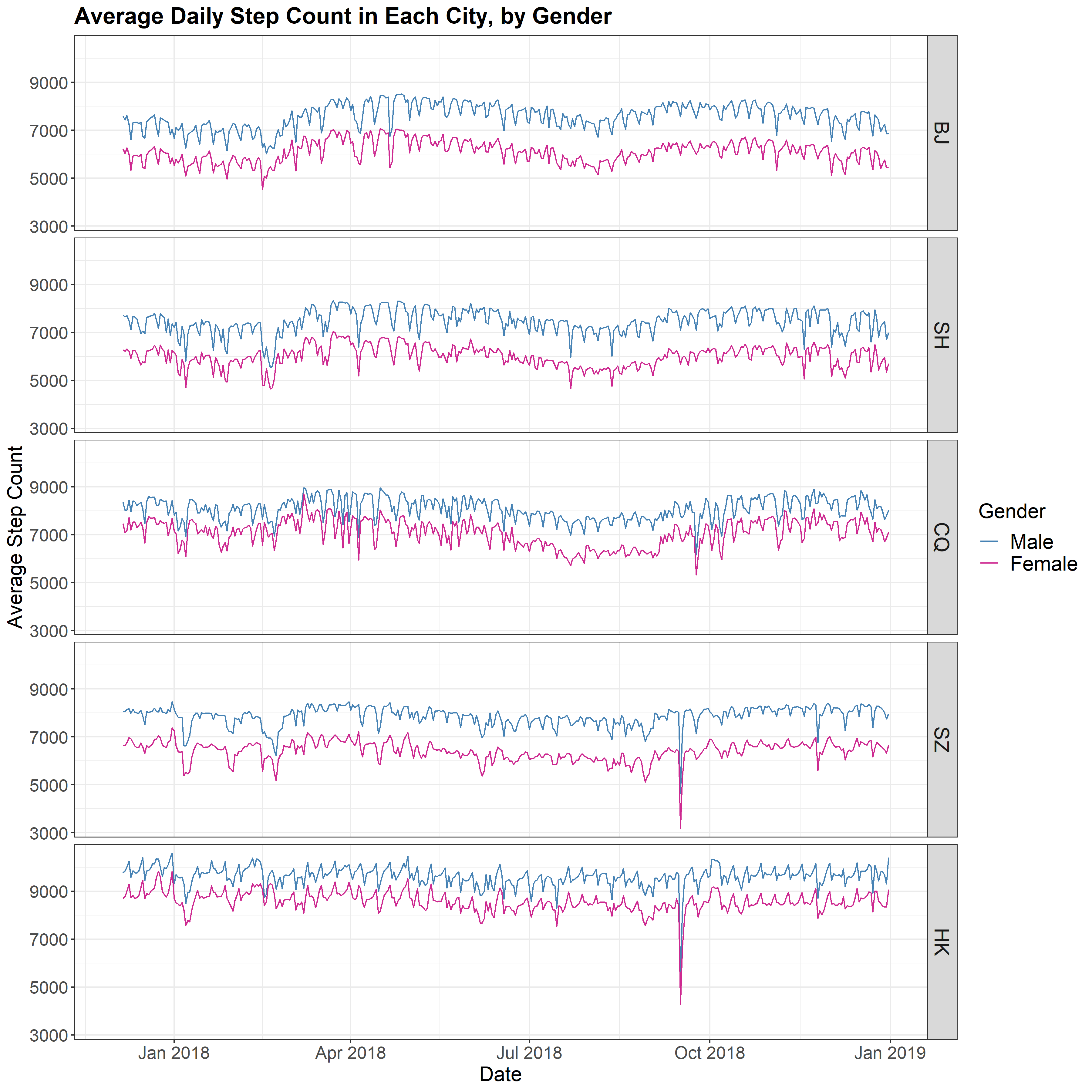
**

**Figure S2.** **Trend of average daily step count in each city, by age**

Note: BJ = Beijing, SH = Shanghai, CQ = Chongqing, SZ = Shenzhen, HK = Hong Kong.

**
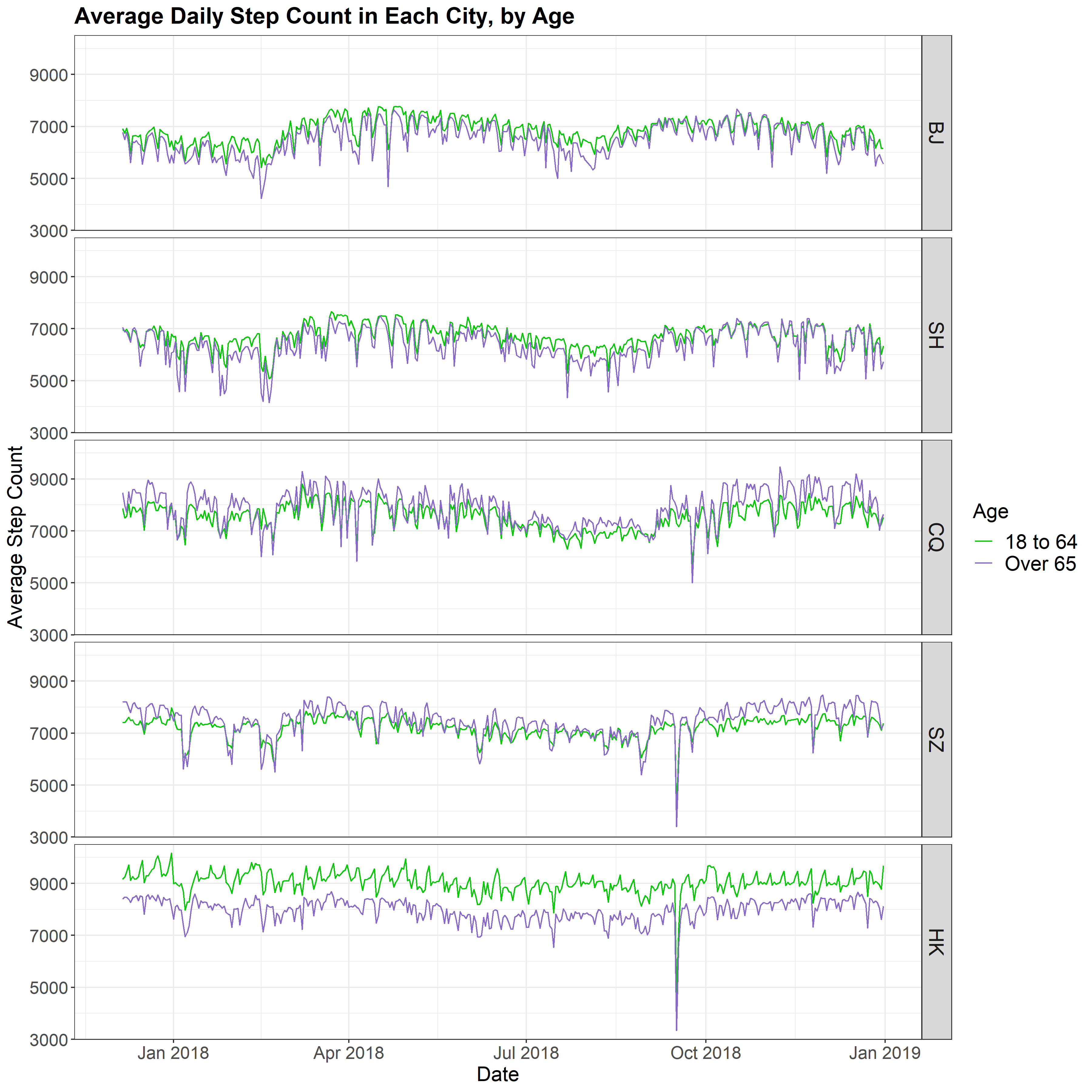
**

**Figure S3. Relationships between other meteorological variables and daily step count in the main models of five Chinese cities** Note: BJ = Beijing, SH = Shanghai, CQ = Chongqing, SZ = Shenzhen, HK = Hong Kong. The model for each city was adjusted for relative humidity#, precipitation, windspeed, pressure#, sunshine, AQI/AQHI, month, day of week, public holiday, extra workdays, typhoon, super typhoon, and marathon (#some cities had these variables removed in the stepdown process). Black markings along the x-axis indicate the actual existing meteorological data of each city; Grey shading indicates the 95% confidence interval.

| BJ | 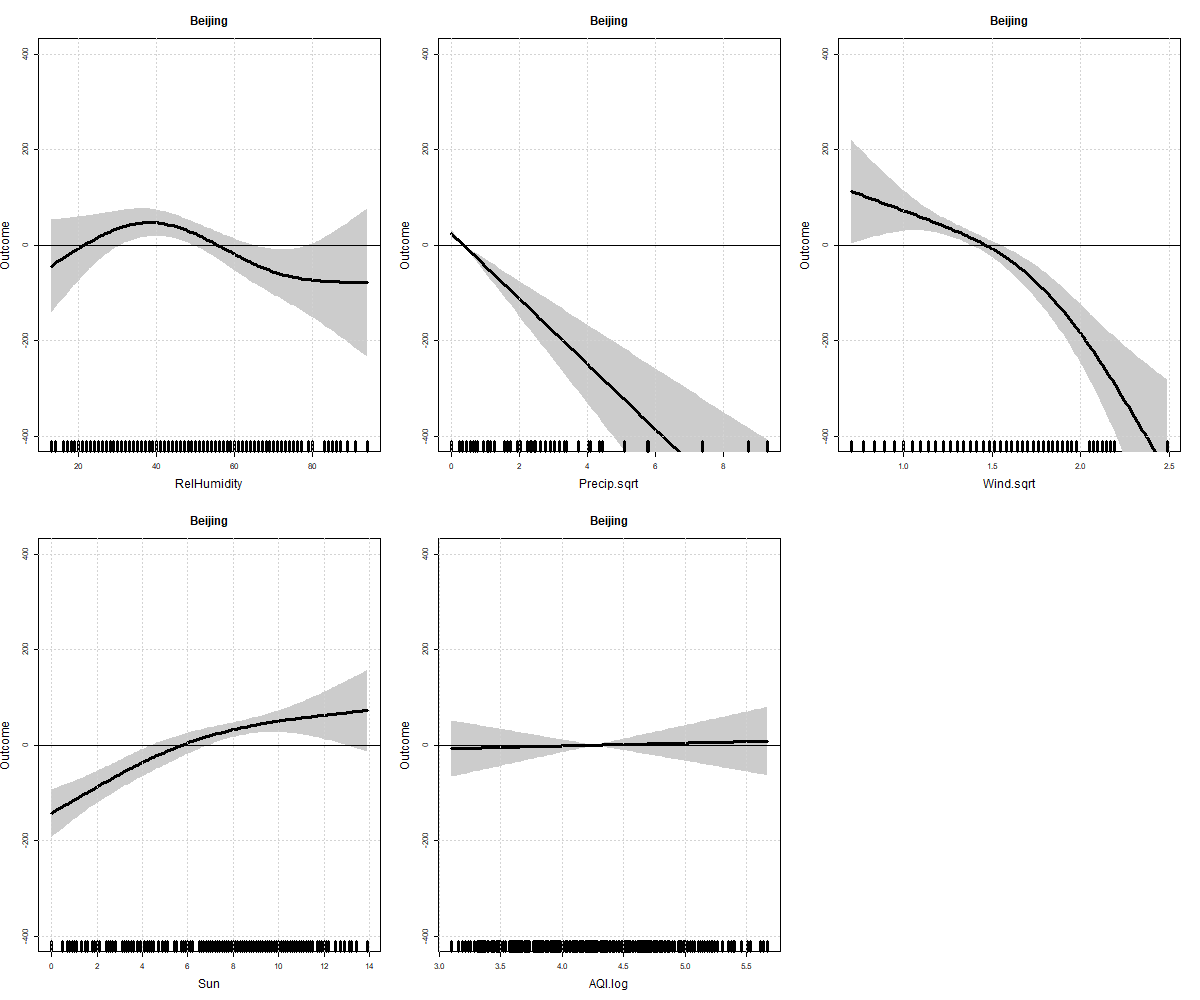 |
| --- | --- |
| SH | 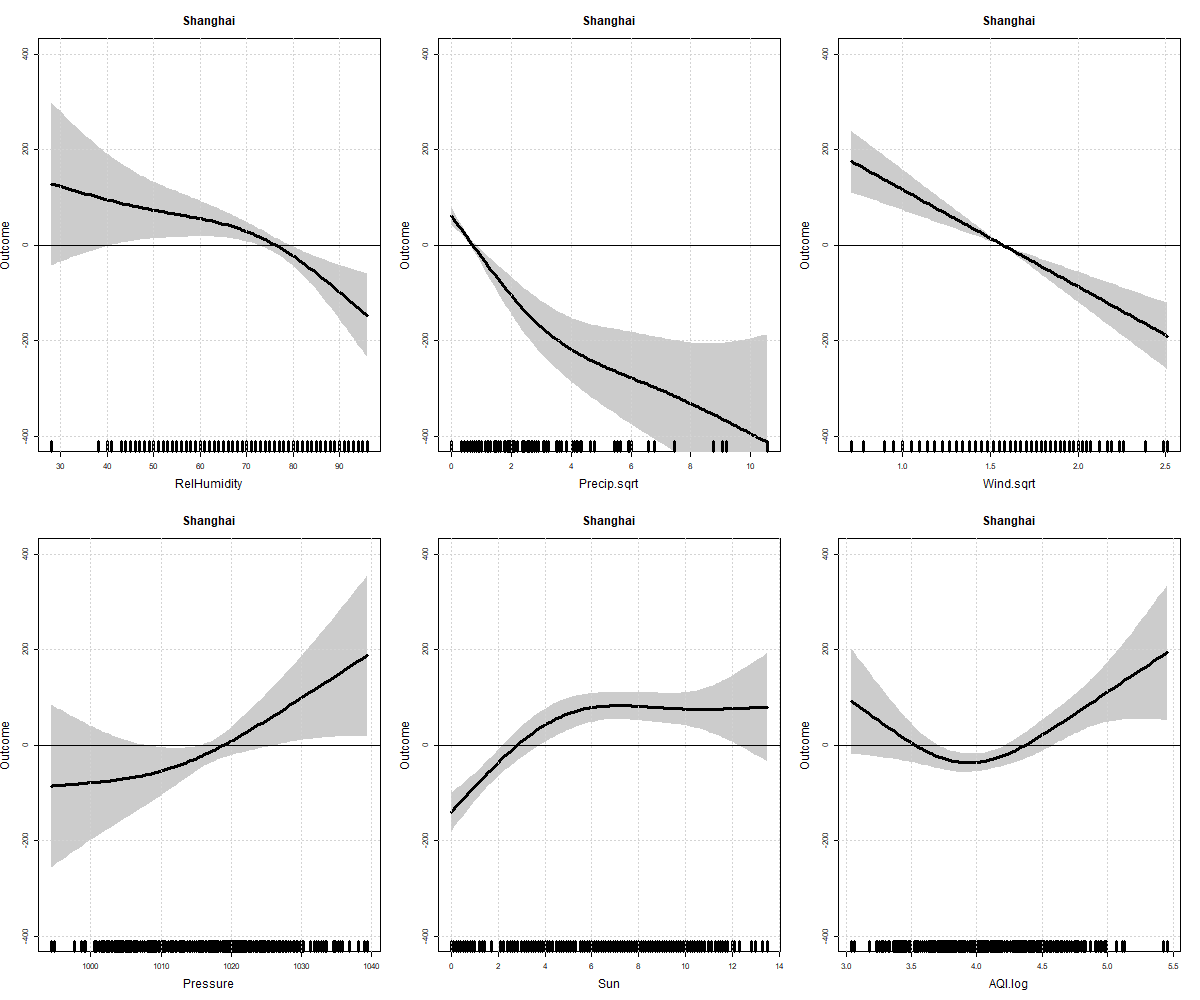 |
| CQ | 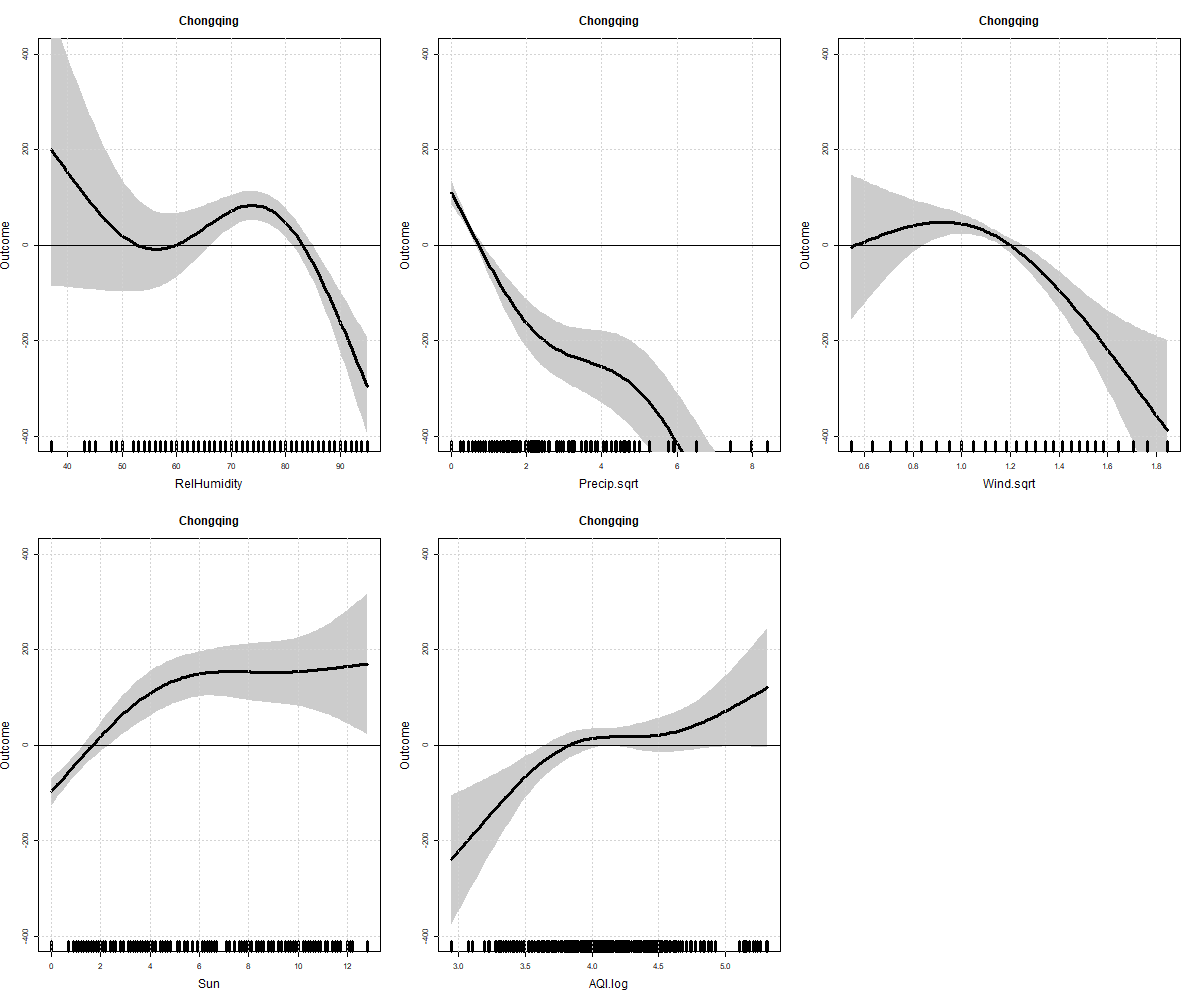 |
| SZ | 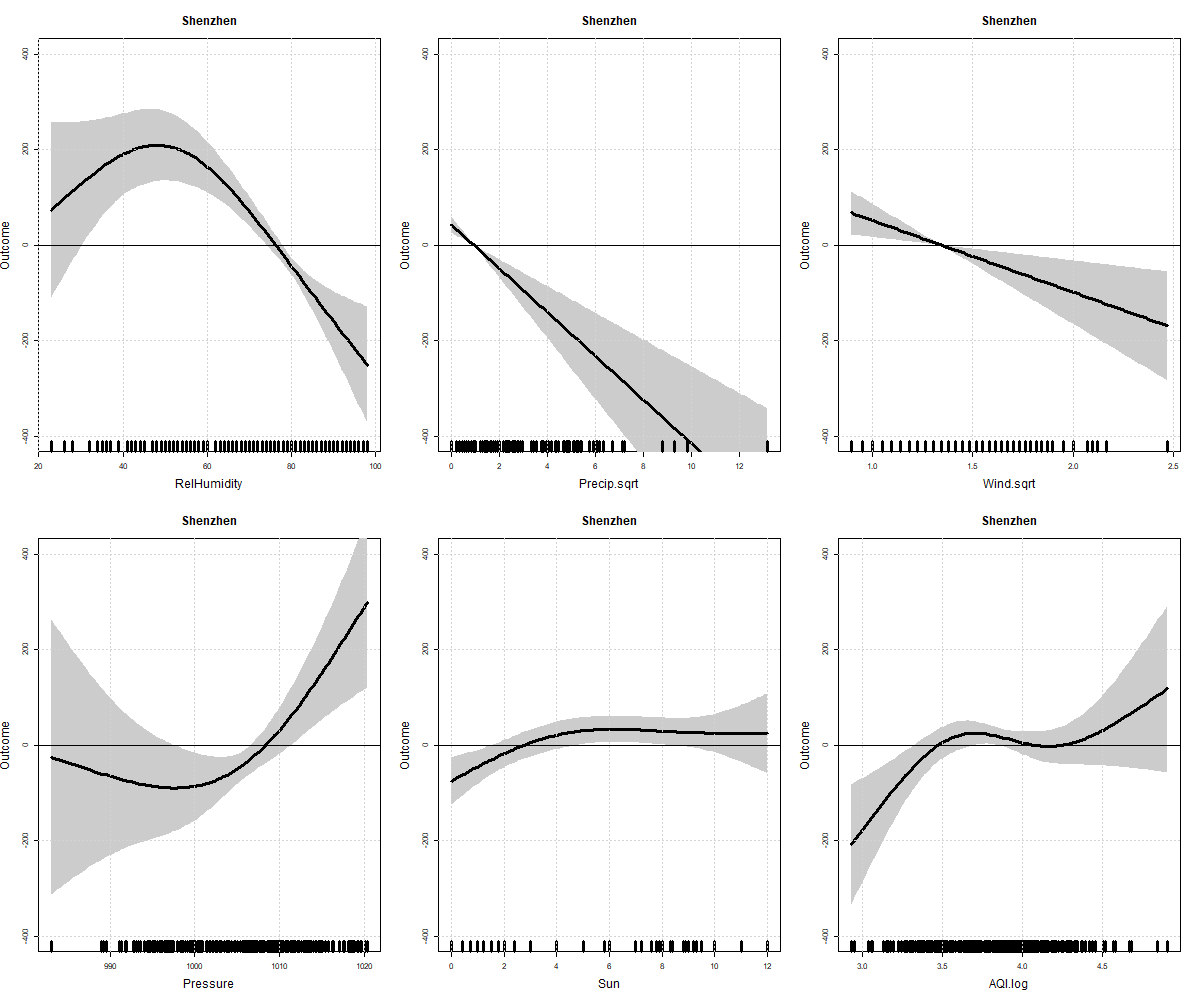 |
| HK | 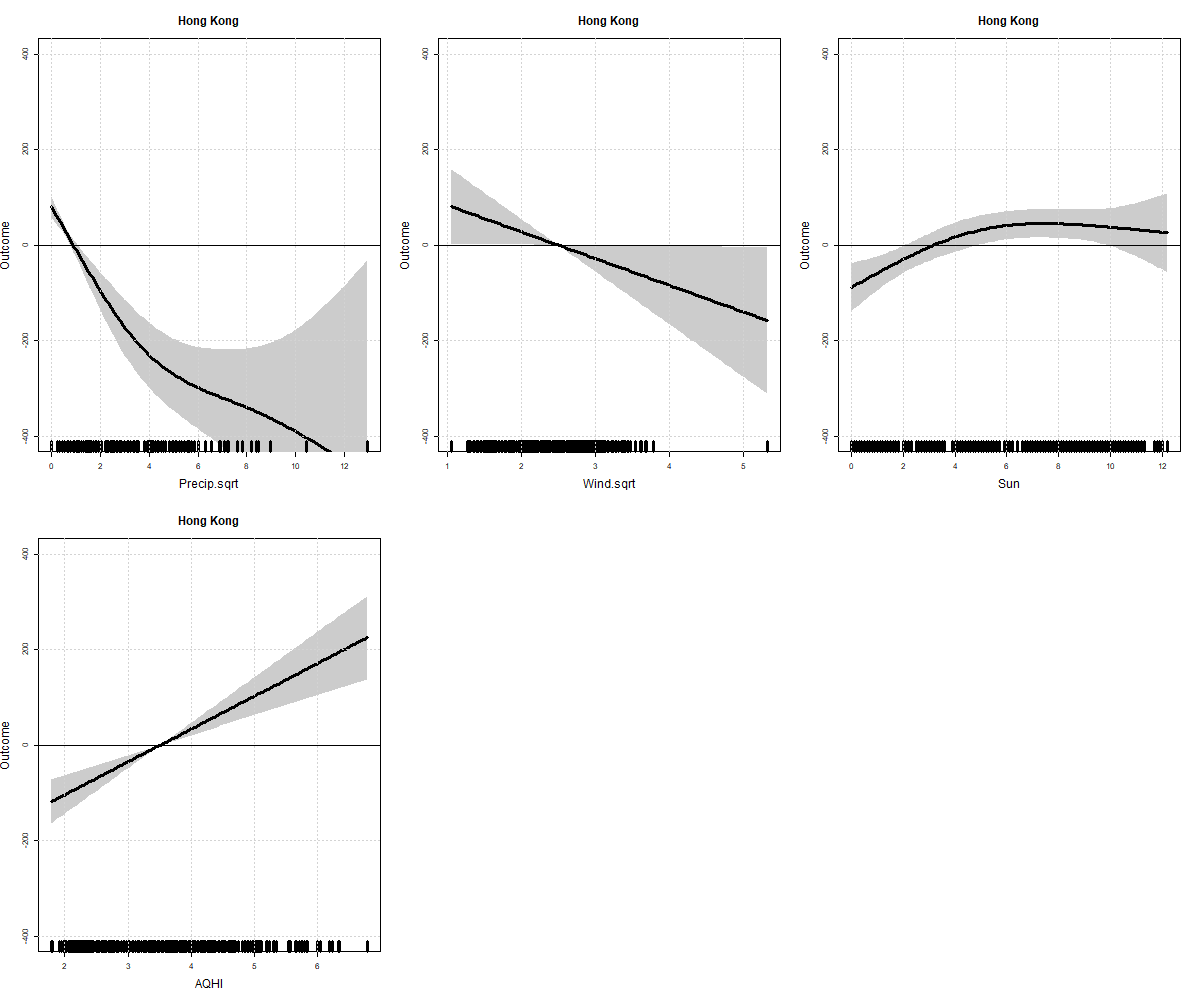 |

| Figure S4. Relationships between temperature and daily step count in five Chinese cities, stratified by gender and age Note: The model for each city was adjusted for relative humidity#, precipitation, windspeed, pressure#, sunshine, AQI/AQHI, month, day of week, public holiday, extra workdays, typhoon, super typhoon, and marathon (#some cities had these variables removed in the stepdown process). Black markings along the x-axis indicate the actual existing meteorological data of each city; Grey shading indicates the 95% confidence interval. |
| --- |
| Beijing  **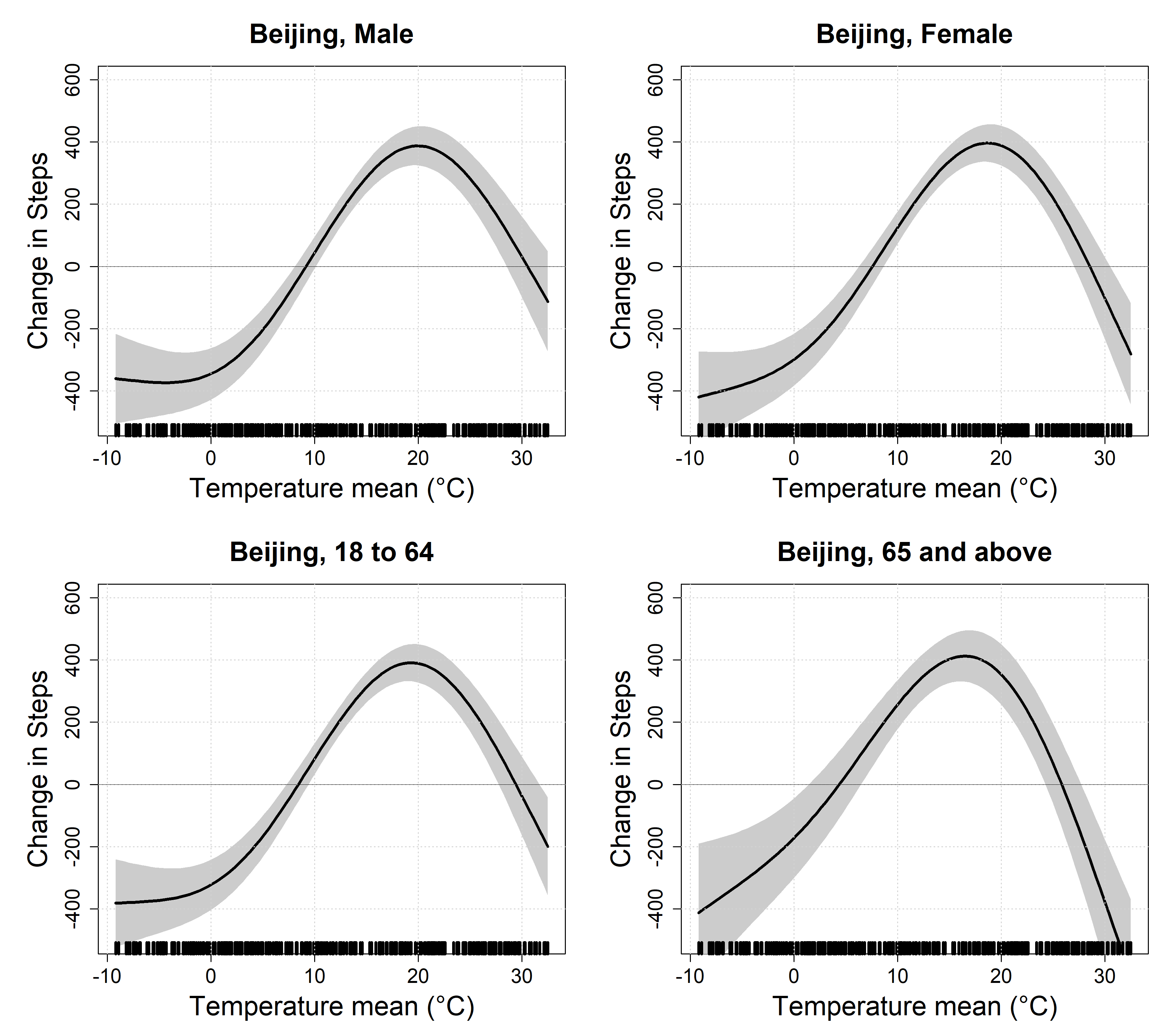** |
| **Shanghai**  **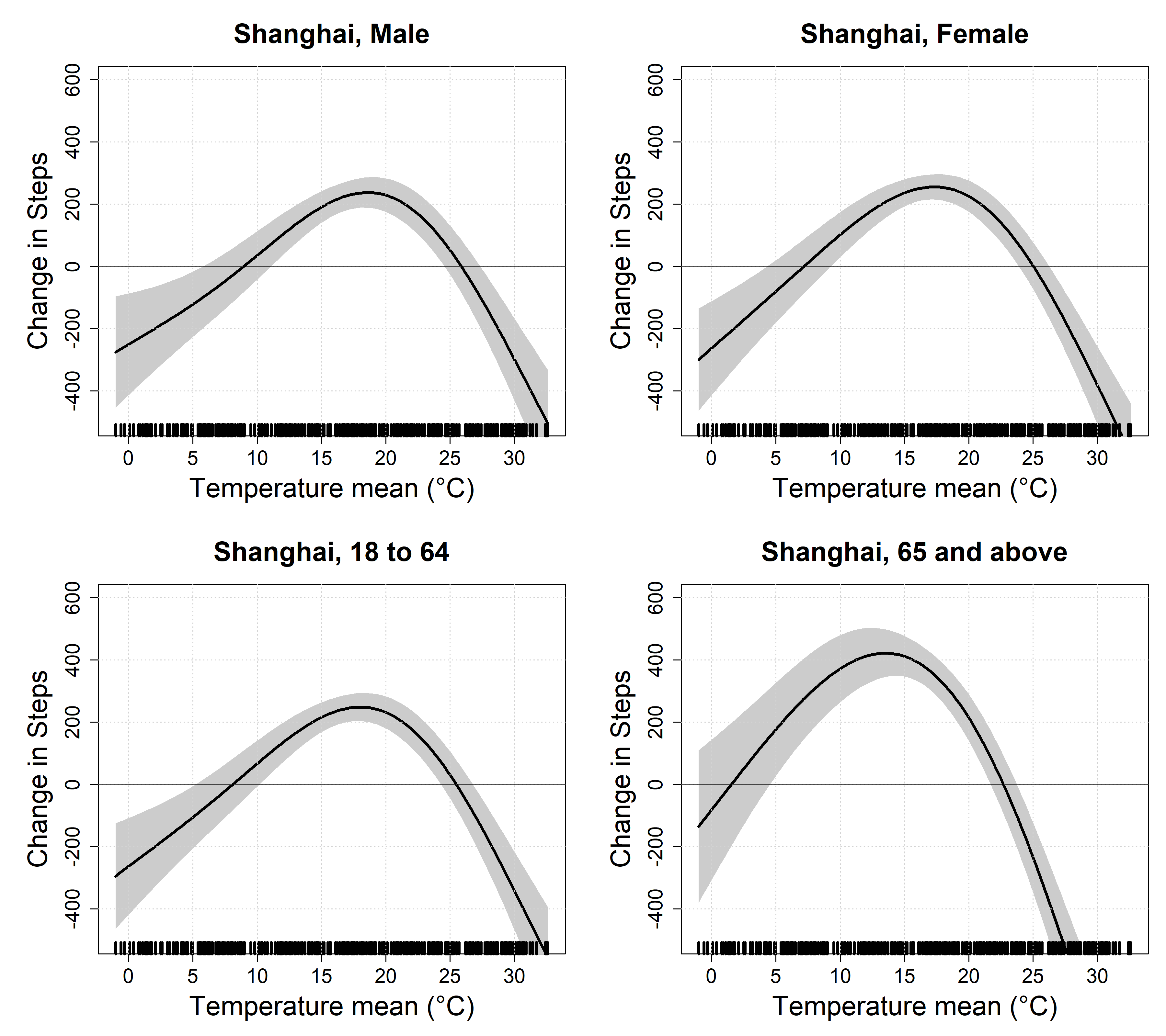** |
| **Chongqing**  **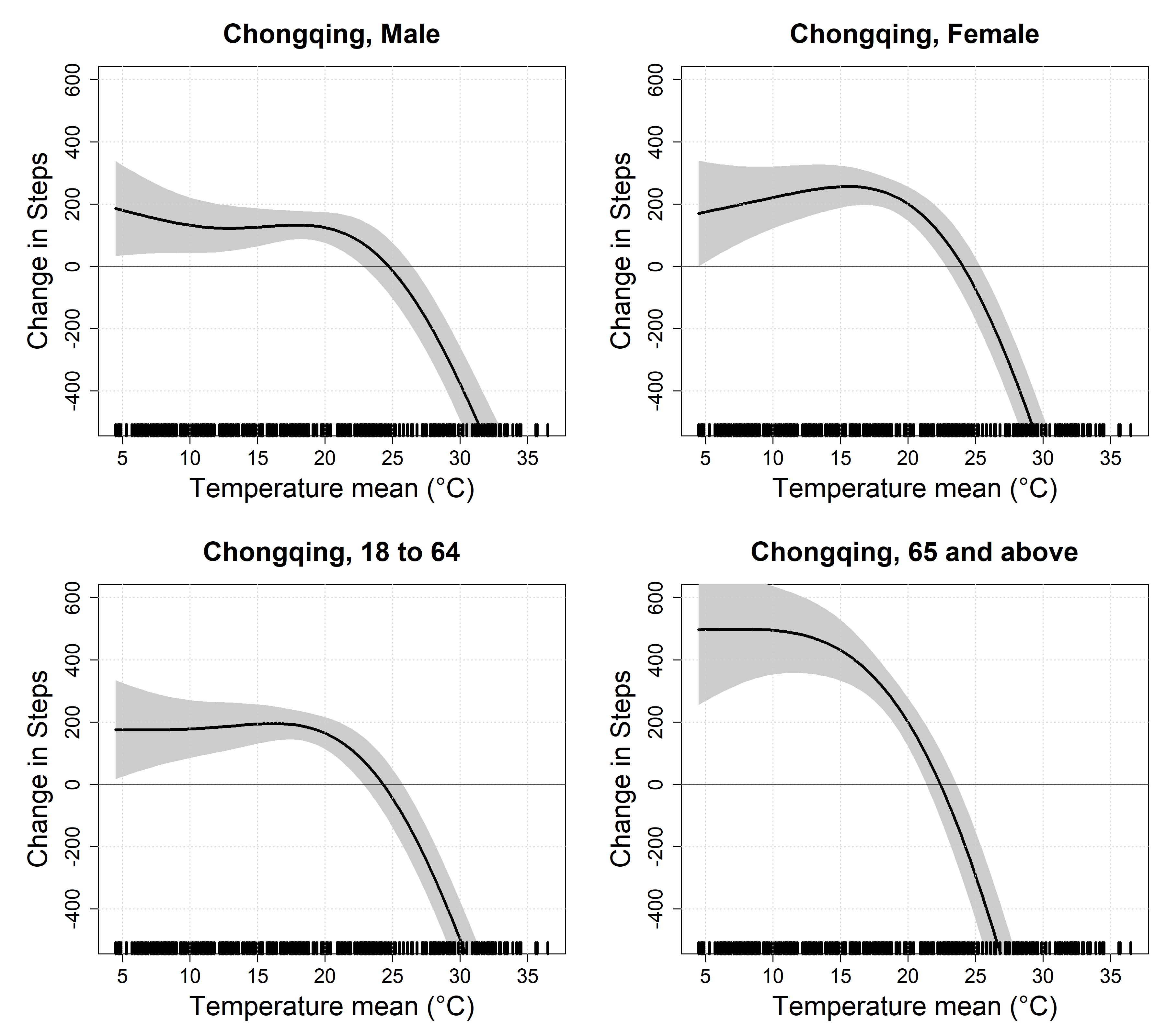** |
| **Shenzhen**  **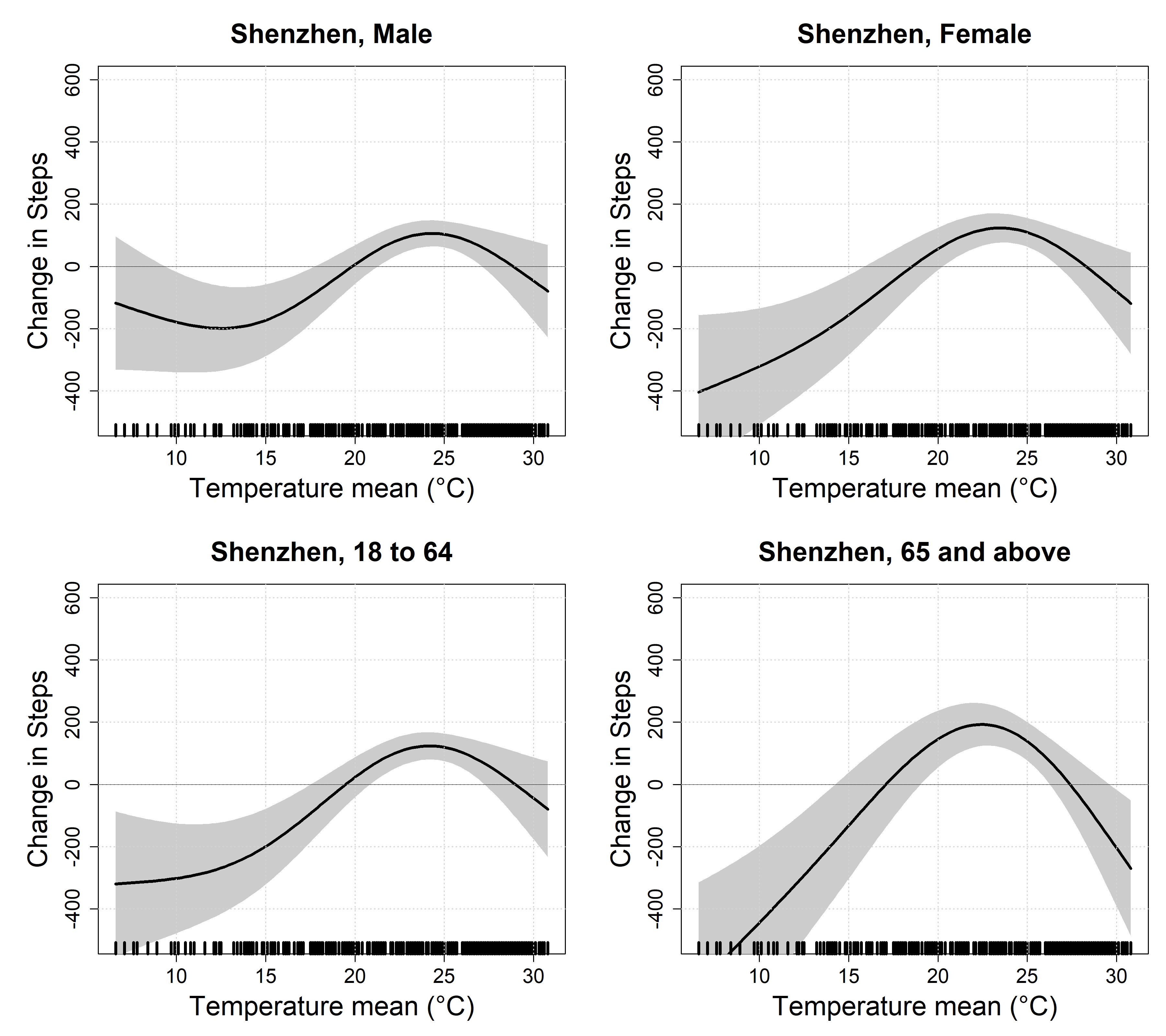** |
| **Hong Kong**  **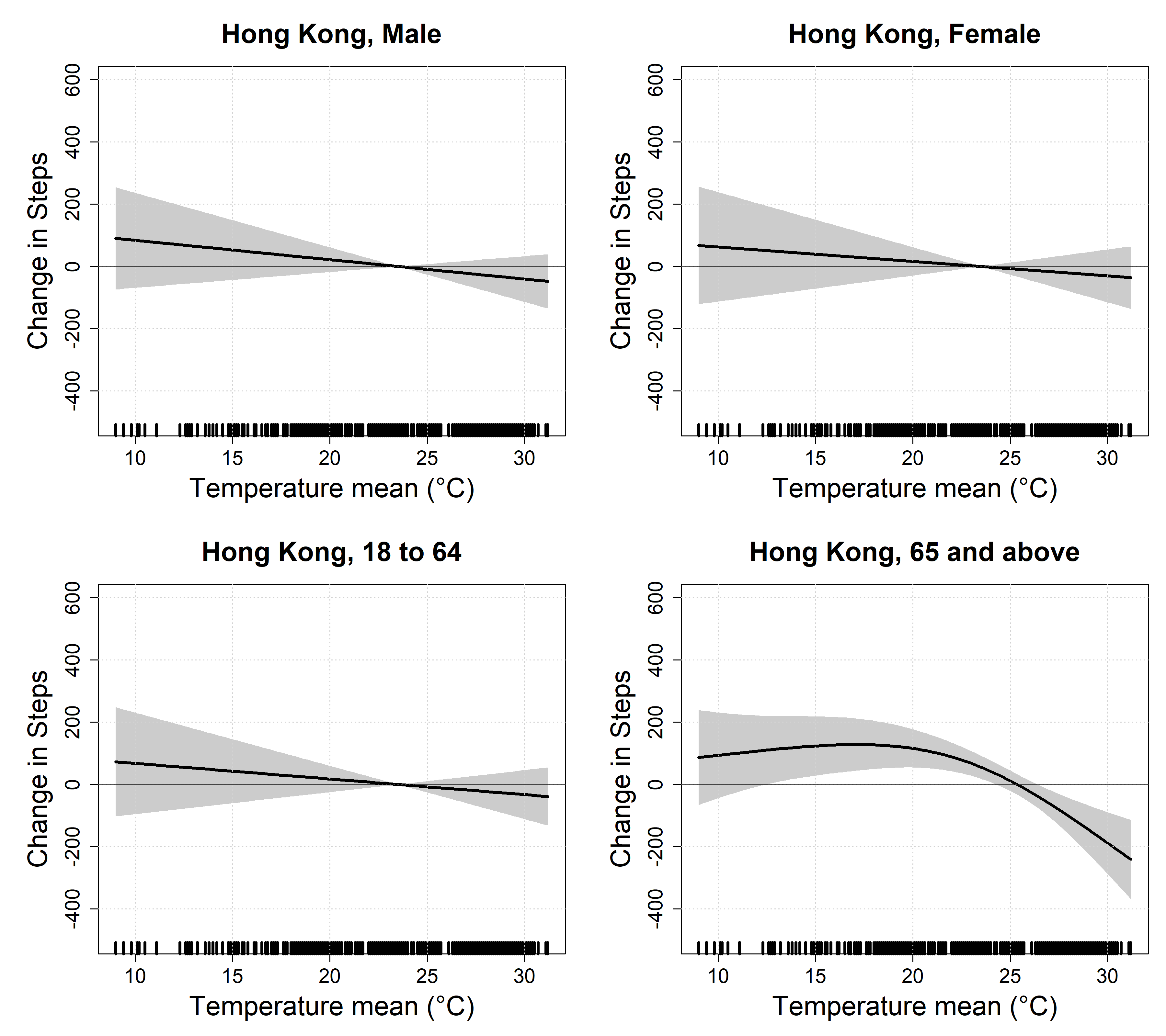** |
